# The effect of crocin on testicular tissue and sperm parameters of mice offspring from mothers exposed to atrazine during pregnancy and lactation periods: An experimental study

**Published:** 2018-08

**Authors:** Masoumeh Fani, Abbas Mohammadipour, Alireza Ebrahimzadeh-Bideskan

**Affiliations:** 1 *Department of Anatomy and Cell Biology, School of Medicine, Mashhad University of Medical Sciences, Mashhad, Iran.*; 2 *Microanatomy Research Center, School of Medicine, Mashhad University of Medical Sciences, Mashhad, Iran.*

**Keywords:** Atrazine, Crocin, Apoptosis, Spermatogenesis, Mouse

## Abstract

**Background:**

Atrazine as a herbicide may affect the human’s health. Crocin may protect atrazine-induced damages.

**Objective:**

The aim of this study was to evaluate the effects of atrazine on mice testicular tissue and sperm parameters and protective effects of Crocin on probably atrazine-induced damages.

**Materials and Methods:**

in this experimental study, 24 pregnant Balb/c mice were randomly divided to 4 groups: I: Atrazine (10 mg/kg), II: Atrazine-Crocin, III: Crocin (10mg/kg) and IV: Normal saline. Administrations were done daily by gavage during pregnancy and lactation. In the end, two male offspring were randomly selected from every mother and sacrificed respectively on 23 and 75 postnatal days. Then, their epididymides were removed for sperm parameters investigation and their testes were prepared to evaluate apoptosis by means of TUNEL technique.

**Results:**

The mean number of sperms in the atrazine group was lower compared to other groups and increased in the atrazine-crocin group compared with atrazine group significantly (p=0.001). Sperm abnormality was increased in the atrazine group compared with the normal saline group and decreased in the atrazine-crocin group compared with atrazine group significantly (p≤0.001). TUNEL-positive spermatogonia in 23 days old offspring increased significantly in the atrazine group compared with other groups (p=0.03). TUNEL-positive spermatogenic cells in 75 days old offspring was significantly increased in the atrazine group compared with the saline group (p≤0.001).

**Conclusion:**

Atrazine exposure may lead to decrease the number of sperms, increase sperms abnormality, spermatogenic cell apoptosis and height of germinal epithelium. These complications may improve by crocin administration.

## Introduction

today, a rising trend is seen in the chemical contamination of the environment by herbicides due to their widespread agricultural use. One of the most widely used herbicides in the world is Atrazine (2-chloro-4-ethylamino-6-isopropyl amino-s-triazine) that is produced from the combination of cyanuric chloride with ethyl amine and isopropyl amine (1). Atrazine was used for the first time in 1958 in order to eliminate weeds from crops (2). In addition to the issue of atrazine absorption by plants, this substance enters surface and underground water resources following its use in agriculture; therefore, atrazine is considered as an important contaminant for surface water and groundwater in most countries because once atrazine is used, it remains in the environment for several years (3-5). 

Many studies have shown that atrazine has adverse effects on various organs of the body, including the brain, kidney, and testis (6-9). Research results show that atrazine can have adverse effects on the reproductive system in both genders. Exposure to atrazine-containing diet in adult women leads to a reduction in the menstrual period time, uterus weight, cytosolic receptors in uterine cells or the early onset of breast tumors. It also reduces the activity of the 5α-reductase enzyme in men that converts testosterone to its active metabolite, 5α dihydrotestosterone, in the anterior pituitary gland and the prostate (10, 11). In addition, atrazine inhibits spermatogenesis and leads to morphological changes in Sertoli and Leydig cells in the testis and weight loss of sexual organs (2). In addition, research results show that atrazine is transmitted from mother to offspring through the placenta or via milk during lactation and then enters various organs of offspring such as testis (9, 12). 

Moreover, the research results show that atrazine also increases oxidative stress and reduces the activity of antioxidant enzymes (6, 13). However, the testicular tissue is very vulnerable to oxidative stress due to the high metabolic activity which can lead to increased apoptosis rate (14, 15) and is characterized with chromatin condensation, DNA fragmentation and eventually cell fragmentation and creation of apoptotic bodies (16). Saffron (Crocus sativus) is a plant of the lily family (17) and has been widely used as herbal medicine, spices, flavoring and food coloring since ancient times. Saffron consists of three main components including Crocin, Picrocrocin, and Safranal (18). Crocin as a carotenoid has antioxidant property. Many studies have shown the therapeutic effects of crocin such as; anti-inflammatory, anti-hypertension, antidepressants, antianxiety, and memory improvement. It also strengthens sexual copulation and prevents cell death caused by oxidative stress (19-21). 

Therefore, considering the harmful effects of atrazine and the transferability of this chemical substance from mother to offspring and the importance of the testis in maintaining healthy generations, this study was designated to evaluate the effects of atrazine on mice testicular tissue, sperm parameters and protective effects of crocin on probably atrazine-induced damages. 

## Materials and methods

**Chemicals and preparation**

Atrazine was purchased from Sigma Co. Crocin was prepared from of Faculty of Pharmacy, Mashhad University of Medical Sciences. TUNEL kit was also purchased from Roche Company, Germany. All drugs were dissolved in normal saline and were given to the mice by gavage. The amount of gavage was adjusted every two days according to the varied weight of each mouse.

**Animals and treatments**

In this study, 24 females and 24 adult male balb/c mice, weighing 30-40 gr were purchased from Animal House of Mashhad University of Medical Sciences and were kept under standard conditions of Animal House (20±2^o^C, 12 hr light/dark cycle). Animals had free access to food and water during the study period. After the adaptation, male and female animals were placed in mating cages with 1:1 ratio. Day zero of pregnancy was determined seeing a vaginal plug. The pregnant mice were later isolated from male mice and kept in separate cages. A total of 24 pregnant mice were randomly divided into four groups:

1. Atrazine group: the animals received 10mg/kg atrazine (22).

2. Atrazine+crocin group: the animals received 10mg/kg atrazine and 10mg/kg crocin.

3. Crocin group: the animals received 10mg/kg crocin (23).

4. Normal saline group: the animals received normal saline.

The administration was started from the sixth day of pregnancy and was carried out up to 23 days after delivery on a daily basis by using the gavage method. At the end of lactation, two male offspring were randomly selected from each mother and one of them at 23rd day and the other one at 75th day after weighting were anesthetized using chloroform and their testis and left epididymis were removed. The removed testis were weighted using scales and placed in buffered formalin 10% for 1 wk.

**Weight index and volume of the testis**

The following formula was used to calculate the weighted index of the testis (Ti):

$\mathrm{Ti}=\frac{\mathrm{Testis weight}(\mathrm{gr})}{\mathrm{animal Body weight}(\mathrm{gr})}\times 100$

Testis volume (Tv) was calculated using the following formula:

$\mathrm{Tv}=\frac{\left(\mathrm{w2}-\mathrm{w1}\right)}{\delta }$

W1=Initial weight (Beaker + normal saline + basket)

W2=Secondary weight (Beaker + normal saline + basket + testis)

δ=Density of normal saline (1.0048)

**Sperm count and morphology**

Removed left epididymis was placed in 1 ml of normal saline and then was cut by scissors and slightly were compressed by a forceps. Afterward, it was diluted by adding 4ml of normal saline and placed in the CO2 incubator set to 37^o^C for 10 min. The mixture was completely homogenized by stirring and one drop was placed on a slide neobar by the sampler. Sperms were counted using an optical microscope with the 40× objective lens. In addition, to investigate the sperm morphology, smears were prepared from this solution and papanicolaou stain was done. Sperm morphology was evaluated on each slide and abnormal sperms (tail-free sperms, sperms with coiled tails or bent tails) were studied and counted using an optical microscope (24).

**Tissue preparation and TUNEL assay**

TUNEL staining was performed to evaluate apoptotic cells in the studied testis tissues. In this method, the samples were fixed in normaline 10% for 1 wk. and dehydrated by ascending degrees of alcohol, cleared using xylene and blocked in paraffin. Paraffin blocks were cut into 5 µm thickness and slides were transferred to Poly-L-Lysine slides. The tissue sections were deparaffinized and were proceed according to routine histological methods. In order to block the tissue internal peroxidase, samples were placed in 3% hydrogen peroxide solution in ethanol for 15 min. After being washed, the samples were incubated with proteinase K for 20 min at room temperature. After washing, the slides were incubated with the reaction solution of the TUNEL staining kit. After washing, the samples were incubated with DAB solution for 15 min at room temperature and then after being washed with PBS the tissue sections were stained with hematoxylin. Finally, cells with brown nucleus were evaluated as TUNEL-positive cells (25, 26).

**Quantification of TUNEL positive cells**

The different regions of the testis were studied and photographed using the microscope camera equipped (Olympus BX51, Japan) with a ×20 objective lens (UPlan FI, Japan). Then images were transferred to a computer and on the computer monitor, the number of apoptotic cells was counted by using rectangular grids placed randomly in the investigated photos. Morphometrically methods were used to count apoptotic cells per unit area in different regions of the testis. Finally, the mean numbers of apoptotic cells per unit area (NA) in different regions of the testis were calculated using the following formula:

$\mathrm{NA}=\frac{\Sigma \overset{\text{®}}{Q}}{a∕f∙\Sigma P}$

Where ∑Q is the number of counted cells in each section, a/f is the area of the frame and ∑P is the sum of the frame associated points hitting the specified space (27).

**The height of the epithelium**

To estimate the height of the epithelium, photos were taken from different parts of testicular tissue using a microscope (Olympus BX51, Japan). The height of the epithelium was later calculated on the monitor screen, using a special Grid and the following formulae:

$\mathrm{Vv}=\frac{\sum_{i=1}^{n}p_{\left(x\right)}}{\sum_{i=1}^{n}p_{\left(\mathrm{total}\right)}}$

Sv=2×∑i=1nIiLP×∑i=1nPi

$H=\frac{V_{v}}{S_{v}}$

In the above-mentioned formula, H is the height of the germinal epithelium, Vv is the volume density of the germinal epithelium and Sv is the surface density of the germinal epithelium. To obtain the surface density (Sv) of the germinal epithelium, the total number of points superimposed on the germinal epithelium (ΣPi), the length of the linear test probe in actual tissue scale (L/P), along with the total number of intersections of linear test probe with the inner surface of the germinal epithelium (ΣIi) were counted. To estimate the volume density of the germinal epithelium (Vv), the total number of points of the point probe superimposed on each image of the testis was counted (ΣPx) and then the total number of points superimposed on the germinal epithelium was also counted and divided into the total number of points counted for the testis (ΣP total).

**Ethical consideration**

This study was conducted at Mashhad University of Medical Sciences, in Mashhad, Iran, and experimental protocols were approved by the Institutional Animal Care and Use Committee.

**Statistical analysis**

The data were analyzed using one-way ANOVA statistical test followed by Tukey post hoc in SPSS software (Statistical Package for the Social Sciences, version 16.0, SPSS Inc, Chicago, Illinois, USA) and p<0.05 was considered significant.

## Results

**Testis weight index**

The results of comparing the weighted index of the testis in 23 and 75 days-old offspring showed that the weighted index of the testis in the atrazine group was decreased compared with other groups, but this decrease was not significant.

**Testis volume**

Comparing the testis volume of 23 and 75-day old mice offspring showed that the volume of testis was decreased in the atrazine group compared to the other groups, but this decrease was not statistically significant.

**Sperms count**

The results showed that the mean number of sperms was significantly decreased in the atrazine group compared to the crocin and normal saline groups (p<0.001). The mean number of sperms was also decreased significantly in the atrazine-crocin group compared to the crocin and normal saline groups (p<0.001). However, the mean number of sperms showed a significant increase in the atrazine-crocin group compared to the atrazine group (p<0.001). There was no significant difference between crocin group and normal saline group in terms of sperm count (Table I).

**Sperm morphology**

Investigating the sperm morphology showed that the percentage of abnormal sperms in the atrazine group was increased significantly compared with both normal saline and crocin groups (p<0.001). In addition, percentage of abnormal sperm showed a significant increase in the atrazine-crocin group, compared to crocin and normal saline groups (p=0.02). The percentage of abnormal sperms in the atrazine-crocin group was significantly lower compared to atrazine group (p<0.001). While, there was no significant difference in the crocin group compared to the normal saline group (Table I).

**TUNEL positive cells assessment**

**Figure 1:**
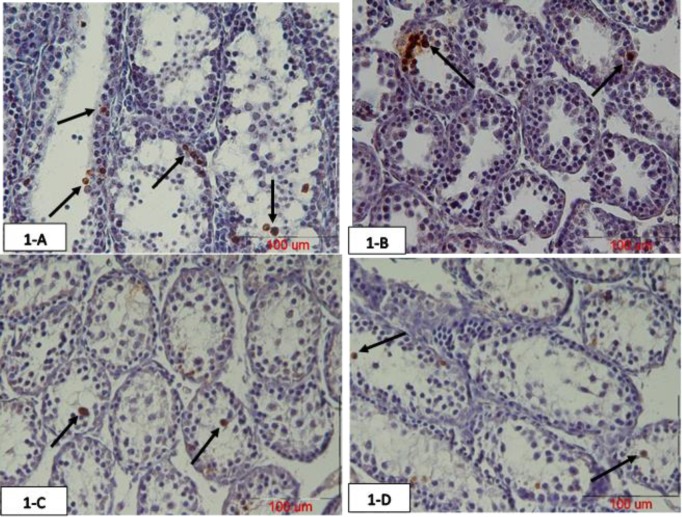
Photomicrograph of testicular tissue of 23 days old offspring of mice prepared using TUNEL staining. Atrazine increased spermatogonia TUNEL positive cells in the testis, while concurrent intake of crocin reduced this TUNEL positive cells. The arrows show TUNEL positive cells

Our result showed that the mean number of TUNEL positive spermatogonia in 23 days old mice offspring was increased significantly in the atrazine group compared with normal saline and crocin groups (p=0.03). Although the number of TUNEL positive cells was decreased in atrazine-crocin group compared to the atrazine group, but this decrease was not statistically significant. The mean number of TUNEL positive cells in the atrazine-crocin group was significantly increased compared to normal saline and crocin groups (p=0.04). There was no significant difference between the crocin group and normal saline group in terms of a mean number of TUNEL positive cells (Figure 1, 5).

**Figure 2:**
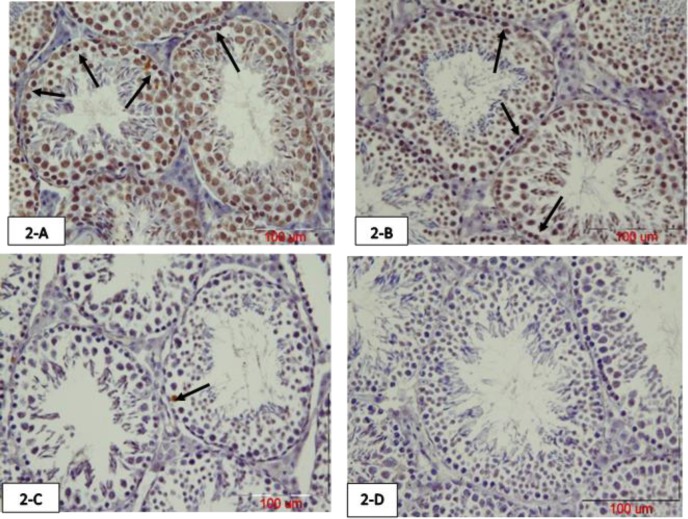
Photomicrograph of testicular tissue of 75 days old offspring prepared using TUNEL staining. The arrows show TUNEL positive spermatogonia cells. Atrazine increased spermatogonia TUNEL positive cells in the testis, while concurrent intake of crocin reduced this TUNEL positive cells

The results of comparing the mean number of TUNEL positive cells in 75 days old mice offspring showed that the mean number of TUNEL positive spermatogonia was significantly increased in the atrazine group compared with normal saline and crocin groups (p<0.001). Although the mean number of these apoptotic cells were increased in the atrazine-crocin group compared to the normal saline and crocin groups (p=0.002) it significantly decreased compared to the atrazine group (p<0.001). There was no significant difference between crocin and normal saline groups in terms of a number of apoptotic cells (Figure 2, 5).

**Figure 3:**
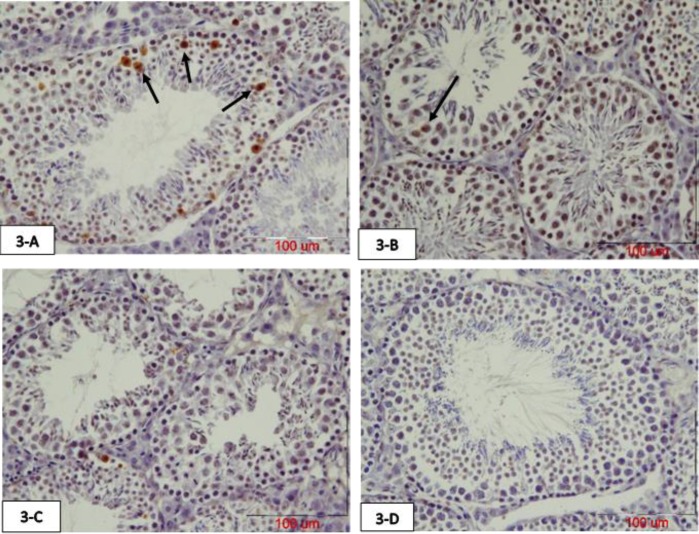
Photomicrograph of testicular tissue of 75 days old offspring of mice prepared with TUNEL staining. The arrows show TUNEL positive primary spermatocyte. Atrazine increased primary spermatocyte TUNEL positive cells in the testis, while concurrent intake of crocin reduced these TUNEL positive cells

**Figure 4:**
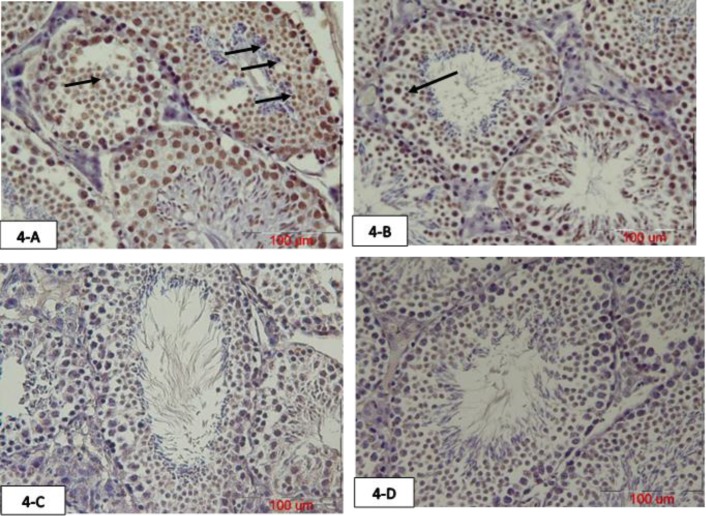
The photomicrograph of testicular tissue of 75 days old offspring prepared with TUNEL staining. Arrows show TUNEL positive spermatids. Atrazine increased spermatid TUNEL positive cells in the testis, while concurrent intake of crocin reduced these cells

**Figure 5:**
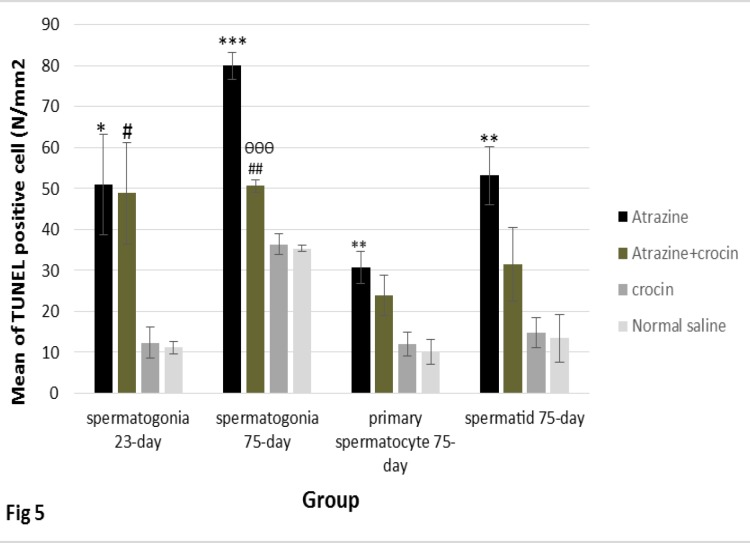
The mean number of TUNEL positive spermatogonia in 23 days old significantly increased in the atrazine (*p<0.05) and atrazine-crocin (^#^p<0.05) groups compared to the normal saline and crocin groups. In 75 days old offspring, the mean number of TUNEL positive spermatogonia significantly increased in atrazine group compared to the normal saline and crocin groups (***p<0.001) and atrazine-crocin group (^##^p<0.01). TUNEL positive spermatogonia significantly decreased in atrazine-crocin group comparing to atrazine group (^ѲѲѲ^ p<0.001). TUNEL positive primary spermatocytes significantly increased in atrazine (**p<0.01) and atrazine-crocin group (&p<0.01) compared to the normal saline and crocin groups. TUNEL positive spermatid increased in atrazine group compared to crocin and normal saline groups significantly (** p<0.001).

The mean number of primary spermatocytes apoptotic cells were significantly increased in the atrazine group compared to the normal saline group (p=0.007) and crocin group (p=0.01). These TUNEL positive cells were decreased in the atrazine-crocin group compared to the atrazine group but this was not significant. There was no significant difference among the other groups (Figure 3, 5). The mean number of TUNEL positive spermatids was significantly increased in the atrazine group compared to normal saline and crocin groups (p=0.003), although the number of TUNEL positive spermatids in the atrazine-crocin group was decreased compared to the atrazine group, but this decrease was not significant. There was no significant difference among the other groups (Figure 4, 5).

**Germinal epithelium height measurement**

**Figure 6:**
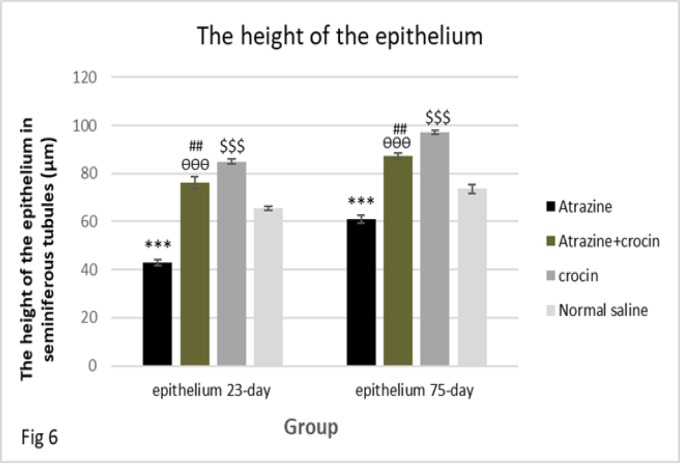
Comparison of the epithelium height in the testis of 23 and 75 days old offspring of mice in different groups. The epithelium height was decreased significantly in the atrazine group compared with normal saline and crocin groups (***p<0.001). Epithelium height significantly increased in the atrazine-crocin group compared to the atrazine (^ѲѲѲ^p<0.001) and significantly decreased compared to the crocin group (^##^p<0.01). Epithelium height was increased in the crocin group compared to the normal saline group (^$$$^p<0.001).

The results of measuring epithelium height in 23 and 75 days old offspring showed that the epithelium height was significantly decreased in the atrazine group comparing to the normal saline and crocin groups (p<0.001). The epithelium height was significantly increased in the atrazine-crocin group compared to the atrazine group (p<0.001) and was significantly decreased compared with the crocin group (p<0.003). In addition, it was significantly increased in the crocin group compared to the normal saline group (p<0.001) (Figure 6).

**Table I:** Different parameters of sperm quality. Data are presented as mean ± SE

**Group**	**Sperm count (10** ^6^ ** per ml)**	**Abnormal sperms (%)**
Atrazine	0.26 ± 0.21 ^a^	^d^45.73 ± 5.1
Atrazine+crocin	1.21 ± 1.89 ^b^^c^	^E^ ^f^24.79 ± 1.6
Crocin	2.47 ± 2.03	11.86 ± 0.6
Normal saline	2.54 ± 1.48	11.85 ± 0.4

a The mean number of sperm increased significantly in atrazine group compared with normal saline and crocin groups ( p<0.001)

b and increased in the atrazine-crocin group compared to the atrazine group ( p<0.001)

c and decreased in the atrazine-crocin group compared to the normal saline and crocin groups ( p<0.001).

d The sperm abnormality percentage increased significantly in atrazine and atrazine-crocin groups compared with normal saline and crocin groups ( p<0.001,

e ( p=0.02 respectively) and decreased compared to atrazine group significantly ( p<0.001).

A: Atrazine group

B: Atrazine-crocin group

C: Crocin group

D: Normal saline group

A: Atrazine group

B: Atrazine-crocin group

C: Crocin group

D: Normal saline group

A: Atrazine group

B: Atrazine-crocin group

C: Crocin group

D: Normal saline group

A: Atrazine group

B: Atrazine-crocin group

C: Crocin group

D: Normal saline group

## Discussion

Atrazine toxicity effects on the human body have been reported but more research is needed about its toxicity mechanisms. On the other hand, Cronin is one of the most important parts of saffron as a powerful antioxidant that can prevent the atrazine toxicity effects (28). In this regard, the present study was designed to examine the effects of atrazine administration during pregnancy and lactation of mice on their offspring testicular tissue and sperm parameters as well as protective effects of crocin on probably atrazine-induced damages.

For determining the atrazine toxicity and protective effects of crocin we assess the weight and volume of the testes, the height of seminiferous epithelium and germinal epithelium apoptosis of 23 (end of lactation) and 75 (sexual puberty) days old male mice offsprings in different studied groups. The results of the present study showed that weight and volume of testis have been decreased in the atrazine group compared with normal saline groups, but this decrease was not statistically significant. There is conflicting information about the impact of atrazine on the weight of testes. For example, results of studies conducted by Aurore Gely-Pernot and colleagues showed that 100mg/kg of atrazine does not affect the weight of the testes (29). 

Another researches were done on the rat model, showed that the exposure to 50 mg/kg of atrazine for 15 days had no effect on testis weight of adult (120 day-olds) rats, while the testis weight was decreased in the rats exposed to 200mg/ kg of atrazine for 40 days (2, 30). In a previous study on 17-20 days old rats, Trentacoste and co-workers observed that only high concentrations of atrazine (100 and 200 mg/kg) for 25 days reduced the weight of androgen-dependent organs such as prostate and seminal vesicles (11). It seems that the impact of atrazine on testis weight is dependent on its concentration and duration of exposure. One of the possible mechanisms for justifying the effect of atrazine is its effect on the 5α-reductase enzyme, converter of testosterone to dihydrotestosterone, which would reduce the concentration of testosterone and weight of androgen-dependent organs, such as prostate and seminal vesicles.

Since testis weight and volume was not affected by the atrazine in the present study, the effectiveness of crocin cannot be determined in this part of the study. However, other researches show that the testicular weight loss was compensated by crocin administration after treatment with nicotine (31). 

In addition, our results showed that atrazine reduced sperm count and increased the percentage of abnormal sperms while crocin reduced these atrazine-induced complications. The results of a study conducted by Gely-Pernot and co-workers showed that atrazine exposure can reduce the count of sperm in the epididymis (29). Previous studies were done on male rats reported that atrazine exposure reduced the sperm count and increased the percentage of the abnormal sperms (2, 32). The results of a research that was done by Najafi and co-*workers* showed that atrazine exposure reduced the count of sperm, sperm viability, and motility (33). It seems that atrazine directly affects spermatogenesis that leads to reduce sperm count and increase morphologically abnormal sperm.

The results of the present study showed that crocin has protective effects and improves some of the atrazine-induced effects on the reduction of sperm count and sperm abnormalities. In this regard, the results of the study conducted by Bakhtiary and colleagues showed that crocin may increase the sperm quality and motility via the reduction of free radicals (24). Also, Salahshoor and co-workers showed that crocin significantly increased sperm quality and motility with its antioxidant effects (31). 

The present study also investigated the apoptotic effects of atrazine on the testicular tissue and the protective effect of crocin. The results of our study showed that atrazine induces germinal epithelium cell apoptosis and co-administration of atrazine and crocin reduces atrazine-induced apoptosis. Therefore, we can obtain a reasonable relationship between apoptosis of spermatogonia cells exposed to atrazine. However, a study on adult mice, Gely-Pernot and co-worker observed no significant changes in the apoptosis rate of Leydig and Sertoli cells. The inconsistency between the results of the above-mentioned study and the present study may be due to the low concentrations of atrazine used (100 mg/L), duration of treatment (2 wk) with atrazine and the mouse species (29).

The height of the germinal epithelium was also assessed in both 23 and 75 days old groups and the results showed that atrazine reduces the height of the epithelium while crocins increases this height. The epithelium of seminiferous tubules with hypoplasia was observed in adult male rats exposed to atrazine in a study on rat model by Farombi and co-workers (2). In another study on adult rats, Song and colleagues showed that in the groups affected by medium and high atrazine doses (38.5 and 77 mg/kg), epithelium of seminiferous tubules was disrupted (32). Atrazine causes toxicity through oxidative stress (6) which is associated with the apoptosis process (34); therefore, it can be concluded that the apoptosis can be an important factor in reducing the thickness of germinal epithelium; while crocin, as an antioxidant, prevents cell death by preventing the formation of free radicals and lipid peroxidation (18).

Generally, it can be suggested that crocin is one of the most important parts of saffron as a powerful antioxidant that can prevent atrazine toxicity effects and DNA damage that may create by oxidative stress (28). In this regard, it may increase sperm count and prevent sperm damage. On the one hand, atrazine leads to inhibition of antioxidants such as catalase and superoxide dismutase and glutathione and on the other hand, increases levels of oxidants such as nitric oxide and malondialdehyde, therefore, active derivatives of oxygen (ROS) accumulate within cells and leads to cell apoptosis through various pathways such as germinal cell DNA fragmentation (6, 13, 35). Testicular tissue is very vulnerable to oxidative stress due to high metabolic activity (15). Pogrmic-Majkic co-worker found that atrazine gavage in rats reduces glutathione transferase, glutathione peroxidase and catalase in testicular interstitial cells (36). 

Thus, it can be concluded that atrazine as a major toxic factor can affect the reproductive system and fertility. In a study on male adult rats, Bakhtiary and colleagues examined the toxic effects of cyclophosphamide and antioxidant effects of crocin on the testicular tissue and part of the results of this study showed that crocin could increase the total antioxidant capacity in blood serum of the above mice simultaneously treated with cyclophosphamide crocin, thus increasing in sperm quality in these animals (24). Bandeghi and co-worker examined the protective effects of crocin in the brain, liver and kidney tissues. The results of their study showed that intraperitoneal injection of crocin reduces oxidative stress factors in these three important tissues (28). 

Another hypothesis for the effect of crocin role to improve atrazine-induced side effects is stimulating LH and FSH and testosterone production, in which may lead to proliferate germinal epithelium cells (30, 37).

## Conclusion

The findings of this study showed that atrazine exposure during pregnancy and lactation can affect the reproductive system and reduce the number of sperms and induce sperm abnormality and germinal epithelium apoptosis. It can also be concluded that crocin as an antioxidant, can greatly reduce the side effects of atrazine and this factor can be used as an effective drug in the future.

## Note

***This article extracted from M.Sc. thesis. (Masoumeh Fani)***
